# Efficacy of Organophosphorus Derivatives Containing Chalcones/Chalcone Semicarbazones Against Fungal Pathogens of Sugarcane

**DOI:** 10.1155/MBD.2002.293

**Published:** 2002

**Authors:** S. K. Sengupta, O. P. Pandey, G. P. Rao, Priyanka Singh

**Affiliations:** 1 Department of Chemistry DDU Gorakhpur University Gorakhpur 273009 India; 2 Sugar Cane Research station Kunraghat Gorakhpur 273008 India

## Abstract

Ten newly synthesized organophosphorus derivatives containing substituted chalcones and substituted chalcone semicarbazones were tested for their antifungal efficacy against *Colletotrichum falcatum*, *Fusarium oxysporum*, *Curvularia pallescens* (all sugarcane pathogens). The O,O-diethylphosphate derivatives containing 2-chlorochalcone and 2-chlorochalcone semicarbazone exhibited 70-85% mycelial inhibition against all the test fungi at 1000 ppm. The screening results were correlated with structural features of the tested compounds.


					Metal Based Drugs Vol. 8, Nr. 5, 2002
EFFICACY OF ORGANOPHOSPHORUS DERIVATIVES CONTAINING
CHALCONES/CHALCONE SEMICARBAZONES AGAINST FUNGAL
PATHOGENS OF SUGARCANE
S.K. Sengupta* ,O.P. Pandey,G.P. Rao2
and Priyanka Singh
Department of Cheoistry, DDU Gorakhpur University; Gorakhpur-273009, India
Sugar Cane Research station, Kunraghat, Gorakhpur 273008, India
ABSTRACT
Ten newly synthesized organophosphorus derivatives containing substituted chalcones and
substituted chalcone semicarbazones were tested for their antifungal efficacy against Colletotrichum
falcatum, Fusarium oxysporum, Curvularia pallescens (all sugarcane pathogens). The O,O-
diethylphosphate derivatives containing 2-chlorochalcone and 2-chlorochalcone semicarbazone exhibited
70-85% mycelial inhibition against all the test fungi at 1000 ppm. The screening results were correlated
with structural features of the tested compounds.
INTRODUCTION
Sugarcane is an important cash crop in many tropical and sub-tropical countries and is one of main sources
of sugar production in the world. The crop is highly susceptible to various fungi, bacteria, viruses
mycoplasma like organisms and other diseases due to which the yield is greatly reduced 2. Because of their
economic importance sugarcane diseases have been studied in great depth from various angles3.
Table I. Physical and Analytical Data ofthe Organophosphorus Derivatives
Cpd Yield Colour Decomp. Moi. Formula
(%) Tern
(C"
Analysis: % Found (Caicd.)
C H N C!
55 Olive green 110 C9Hz2OsP 66.3 (66.5) 6.0
(6.1)
II 62 Yellow 105 C9H22OsP 66.2 (66.5) 6.0
brown (6.1)
III 51 Dark brown 110 CI9H2206P 63.2 (63.5) 5.6
(5.8)
IV 57 Dark yellow 70 CgHzlOsCIP 60.4 (60.5) 5.1(5.3)
V 60 Light brown 47 C20H2406P 53.8 (54.0) 5.0
(5.)
Vl 58 Dark brown Semi C_oH25OsN3P 57.9 (58.0) 5.6 9.9
solid (5.8) (10.0)
VII 55 Olive green 80 C2oH2505 N3P 57.8 (58.0) 5.6 9.7
(5.8) (o.o)
VIII 59 Dark brown 110 C24H3509 N3P2 55.4 (55.6) 5.4 9.6
(5.6) (9.7)
IX 55 Yellow 95 C2oH2405 N3CIP 53.2 (53.3) 5.0 9.1(9.
(5.) )
7.7
(7.8)
X 60 Dark brown Semi C21H2706 N3P 56.4 (53.3) 5.7 9.3(9.
solid _(-8) 4)
293
S.K. Sengupta et al. Efficacy ofOrganophosphorusderivatives Containing Chalcones/
Chalcone Semicarbazones against Fungal Pathogens ofSugarcane
A number of synthetic organic compounds viz., dithiocarbamates, carbamates, organochlorine,
organomercuriale, thiocarbamates and hydrazides are now known to be useful in the control of various
fungal diseases in plants45. Currently we are engaged6"11
in synthesizing novel organophosphorus
derivatives, which could constitute a new and promising field of application in the national economy. It
was realized that on the basis of suitable logic, organic molecules incorporating phosphorus might be
designed such that they may be less dangerous in use without losing thesis value as effective pesticides.
The present study was therefore undertaken to evaluate the antifungal efficacy of some newly synthesized
organophosphorus compounds against various important fungal pathogens of sugarcane.
Table II. IR Spectral Data (cm of Organophosphorus Derivatives
Compound v (C=O) v (C=N) v (P-O-C) v (P=O)
1685 s 1035 m 1290 m
II 1665 s 1035 m 1290 m
III 1670 s 1025 m 1280 m
IV 1675 s 1030 m 1275 m
V 1680 s 1030 m 1265 m
VI 1700 s 1525 s 1020 m 1270 s
VI! 1670 s 1485 s 1025 m 1275 s
VIII 1680 s 1475 s 1025 m 1270 s
IX 1700 s 1510 s 1015 m 1275 s
X 1685 s 1500 s 1020 m 1265 s
EXPERIMENTAL
The reactions of O,O-diethylchiorophosphate were carried out under inert atmosphere and
anhydrous conditions. Special precaution were taken to exclude moisture from the apparatus and chemicals
as the starting materials (O,O-diethylchlorophosphate) and reactions were susceptible to hydrolysis. Glass
apparatus with interchangeable joints were used throughout the work. The solvents were purified and dried
using the method described in the literature2. O,O-diethylchlorophosphate was prepared according to the
reported method 3. Chalcones/chalcone semicarbazones were prepared as described4. All reactions were
carried out in the hood. A hood is a specially constructed workplace that has, at the least, a powered went to
suck noxious fumes outside. The details of analysis and physical measurements were the same as reported
earlier9.
For antifungal activity all the compounds were tested against all the test fungi by the food poison
technique 5
at three concentrations (10, 100, 1000 ppm). For this the desired amount of chemical was
dissolved in 0.5 cm of solvent and mixed with the culture medium on the basis of the volume of medium in
each petriplate (80-ram diameter). Oat meal agar medium 6
was used for all test fungi. In controls, the
same amount of medium containing the requisite amount of solvent was poured in place of test chemicals.
A mycelial disk (5-ram diameter) obtained from the periphery of 2 week old cultures was taken and
transferred to the center of each petriplate. Plates were incubated for 7 days at 28 +-2o
C. Each treatment
was repeated three times and the inhibition was a recorded relative to percent mycelial inhibition calculated
using the formula.
[(dC-dT)/dC x 100
where dC is the average diameter of the mycelial colony of the control and dT is the average diameter of
the myceliai colony of the treatment.
Synthesis of Organophosphorus derivatives
A mixture of O,O-diethyl chlorophosphate (10 mmol) and the substituted chalcone
semicarbazones of substituted chalcones (10 retool) were refluxed in ethyl alcohol (40 cm3 in presence of
pyridine (5 cm for about 30- 40 hours The reaction mixture was cooled and poured in ice. The
precipitate, thus obtained, was filtered off. The compound was recrystallised from ethanol.
294
Metal Based Drugs Vol. 8, Nr. 5, 2002
(C'---CH3 OCH
ethanol
C--CH=C
-H20"-
2-Hydroxybenzal
acetophenone (BDAH)
O
(C2HsO)2P-CI
O
C---CH=CH
O
Ct-IsO
CHsOP=O
(C2HsO)2PO(BDH)
Scheme 1" Synthetic route ofO,O-diethyiphosphate derivative containing 2-hydroxy chalcone
(CHO CH3-(
O
OH
NaOH, C2HsOH
1
_
-'H20 -'CH=CH
O
Benzal- 2'-hydroxy
acetophenone (BCAH)
Ethanol
pyridine
C'HsO'P=O
O
O
C2H
P-CI
O
C--CH=CH
(C2HsO)2PO(BCA)
Scheme 2: Synthetic route ofO,O-diethyl phosphate derivative containing 2'-hydroxy chalcone
295
& K. Sengupta el al.
H
C-- CH3
Efficacy ofOrganophosphorusderivatives Containing Chalcones/
Chalcone Semicarbazones against Fungal Pathogens ofSugarcane
HCI, NaOH OH
_
ethanol
C--CH=C
-H20
2,2'-Dihydroxybenzal
acetophenone (BDAH2)
O
C2H5C
2
C2HC P-CI
CHsO,
CHO, P=O P=O
CiHsO/IO O
[{(C2HsO)2PO}2 (DBA)]
Scheme 3" Synthetic route ofO,O-diethylphosphate derivative containing 2,2'-dihydroxy chalcone
OH
C--CH3
HCI, NaOH OH CI
ethanol
-H20
> CH=CH
2-chlorobenzal-2'-hydroxy
acetophenone (CBAH)
O
C2Hs%II
C2HO
P-CI
C2HsC P=O
C2HsO
O
C-- CH=CH
[(C2HsO)2PO (CBA)]
Scheme 4: Synthetic route ofO,O-diethyl phosphate derivative containing 2-chloro-2'-hydroxy chalcone
296
Metal Based Drugs Vo/. 8, Nr. 5, 2002
RESULTS AND DISCUSSION
The reactions of O,O-diethylchiorphosphate with substituted chalcones derived by the
condensation of 2-hydroxybenzaidehyde and acetophenone; benzaldehyde and 2-hydroxybenzaldehyde; 2-
hydroxybenzladehyde and 2-hydroxyacetophenone; 2-chlorobenzaidebyde and 2-hydroxybenzaldehyde or
3-methoxybenzaldehyde and 2-hydroxyacetophenone, have been carried out in ethanol in the presence of
pyridine and a variety of organophosphorus derivatives (types V have been isolated according to
Schemes 1- 5.
Table III. HNMR 05, ppm) Data of Organophosphorus Derivatives
Cpd -CH CH CH3 CzHs NH -NH2
6.82 (d), 5.75 (d) 2.75 (t), 3.38 (q)
II 6.95 (d), 5.88 (d) 2.60 (t),3.00 (q)
III 7.00 (d), 5.82 (d) 2.80 (t), 3.40 (q)
IV 6.85 (d), 5.90 (d) 2.65 (t), 3.26 (q)
V 6.80 (d), 5.86 (d) 1.95(s) 2.72 (t),3.35 (q)
VI 4.60(d),5.35(d) 2.45 (t), 3.28 (q) 5.40(s) 6.45 (s)
VII 4.75 (d), 5.55 (d) 2.55 (t), 3.30 (q) 5.48 (s) 6.40 (s)
VIII 4.65 (d), 5.00 (d) 2.60 (t), 3.10 (q) 5.45(s) 6.48 (s)
IX 4.72 (d), 5.48 (d) 2.50 (t), 3.25 (q) 5.50 (s) 6.38 (s)
X 4.70 (d), 5.28 (d) 1.96 (s) 2.58 (t), 3.15 (q) 5.42 (s) 6.50 (s)
The reactions of O,O-diethylchlorphosphate with semicarbazones of substituted chalcones
derived by the condensation of 2-hydroxybenzalacetophenone benzal -2'- hydroxyacetophenone/2,2'-
dihydroxybenzalacetophenone/ 2-chlorobenzal 2-hydroxyacetophenone 3-methoxybenzal-2'-
hydroxyacetophenone and semicarbazide have been carried out in ethanol in the presence of pyridine and a
variety of organophosphorus derivatives (types VI- X have been isolated according to Schemes 6 10.
The analytical data and physical properties of all organophosphorus derivatives are given in
Table-i. The methods used for the preparation and isolation of these compounds give materials of good
purity as supported by their analysis and TLC. The spectral (IR, H NMR) data are given in Tables 2 and
3.
Anti fungai activity
Results of the antifungal assay of the organophosphorus derivatives are summarized in Table 4.
The compounds were screened for their antifungal properties against Colletotrichum falcatum, Fusarium
oxysporum, and Curvularia pallescens (all parasitic on sugarcane). Organophosphorus derivatives
containing substituted chalcones showed promising results in inhibiting the mycelial growth of all the test
fungi. The derivatives containing 2-chlorobenzal-2'-hydroxyacetophenone (IV) showed inhibition upto 84
% for C. falcatum and 80.2% for F. oxysporum at 1000 ppm concentration. Other derivatives showed
inhibition 55.1 to 80.3% against all test fungi at 1000 ppm concentration.
The derivatives containing semicarbazones of substituted chalcones were found to be less active
than substituted chalcones.
297
S.K. Sengupta et al. Efficacy ofOrganophosphorusderivatives Containing Chalcones/
Chalcone Semicarbazones against Fungai Pathogens ofSugarcane
Table 4. Fungitoxic Screening Data ofOrganophosphorus Derivatives
Compound Percent Mycelial Inhibition
Compound Dose (ppm)
Colletotrichumfalcatum
10 100
1000
Fusarium oaysporum
10 100 1000
Cma,ularia pallescens
10 O0 1000
40.18 58.6 78.0 31.5 44.2 68.1 30.5 44.7 65.2
II 28.6 35.6 80.1 20.0 43.2 70.3 11.6 33.3 55.1
III 34.5 60.8 80.3 30.3 50.9 75.6 47.2 51.8 69.8
IV 27.6 50.4 84.0 33.3 60.6 80.2 21.7 45.9 74.2
V 25.3 40.6 80.1 22.1 50.2 78.3 12.7 50.2 68.6
VI 18.8 40.9 71.2 10.6 37.8 59.2 12.1 30.3 55.1
VII 10.7 48.2 68.1 16.7 44.3 60.6 15.0 39.8 50.3
VIII 22.8 45.2 72.1 20.1 41.3 64.2 20.2 40.3 61.2
IX 25.5 49.7 73.0 22.4 46.0 62.1 15.6 39.8 62.0
X 18.7 34.6 63.4 18.2 26.2 63.8 13.2 22.2 57.7
OH OCH3 HCI, NaOH
ethanol
lH
..OCH3
> C--CH=CH
-H20
3-Methoxybenzal-2'-hydroxy
acetophenone (MBAH)
O
C_I-t50 II
C2H50,/ P-CI
C2H50, P=O
C2H50,
O
C'-"
CH=CH-OCH3
Scheme 5"
(C2HsO)2PO (MBA)
Synthetic route of O,O-diethyl phosphate derivative
3-methoxybenzal-2'-hydroxyacetophenone (MBAH)
containing
298
Metal Based Drugs Voi. 8, Nr. 5, 2002
0 0
II
HN.NH _.-NH:HCI + CHsCOONa
CH-CH-C +
reflux
methanol_H20
O
H
II-NH-C-NH2
CH=CH-C + CH3COOH + NaCI
2-Hydroxybenzalacetophenone semicarbazone (BASH)
ethanol C2H5 II
pyridine C2Hs
P-CI
C2H50"
p=O O
C2H50
N-NH-C-NH2
II
CH-CH-C
-'
[(C2HsO)2PO (BAS)]
Scheme 6: Synthetic route of O,O-diethyl phosphate derivative containing 2-hydroxy chalcone
semicarbazone.
o
H=CH-C +
reflux
methanol_H20
O
H
IN-NH-C-NH2
CH=CH-C + CHBCOOH + NaCI
Benzal,2'-hydroxy benzalacetophenone semicarbazone (BDSH)
ethanol
pyridine
O
C*H5>II
P-CI
C2H5
O
II
N-NH-C-NH
CH=CH-C
O
CHsO
CHsO P=O
[(C2HsO)2PO (BAS)]
Scheme 7: Synthetic route of O,O-diethyl phosphate derivative containing 2'-hydroxy chalcone
semicarbazone.
299
S.K. Sengtqta et E.[.]k'ac3' q["Organophosphorusderivatives Containing Chalcones/
Chalcone Semicarbazones against Fungal Pathogens ofSugarcane
methanol
-HO
H-C-NH
CH=CH-C- + CHCOOH + NaCI
H
2.2'-Dihydroxybenzalacetophenone semicarbazone (DBSH2)
ethanol
vC2H5%_
t3vridine C2H50" CI
N-NH-C-NH_
C HsO
P=O O= OC2H5
OC2H5
[(C2HsO)2PO (DBS)]
Scheme 8: Synthetic route of O,O-diethyl phosphate derivative containing 2,2'-dihydrox3
chalcone semicarbazone.
+ H2$NH-C-NH2HCI+
methanol
-NH-C-NH2
2H=CH-C- + CH3COOH + NaCI
2,2'-Chlorobenzal,2'-hydroxyacetophenone semicarbazone (CBSH)
ethanol / CHsO)
tvridine CzHO"P-CI
N-NH-C-NH2
O=pOC2H5
OC2H5
[(C2HsOhPO (CBS)I
Scheme 9: Synthetic route of O,O-diethyl phosphate derivative containing 2-chloro.
2'-hydroxy chalcone semicarbazone.
3OO
Metal Based Drugs Vo/. 8, Nr. 5, 2002
0
OH3C HO,
reflux methanol_H20
o
N-NH-C-NH
CH=CH-C + CH3COOH + NaCI
OH3CI
O
+ H2N.NH{-NH2HCI + CH3COONa
3-Methoxy benzal- 2'-hydroxyacetophenone semicarbazone (MBSH)
ethanol C)Hs II
pyridine CHs P-CI
O
N-NH-C-NH2
CH=CH-C
OHsC O
O=;<OC,H
OCH
[(C2HsO)2PO (MBS)]
Scheme 10: Synthetic route of O,O-diethyl phosphate derivative containing 3-methoxy-2'-hydrox)
chalcone semicarbazone.
The best activity was recorded with O,O-diethylchlorophosphate derivative containing 2-
chiorobenzal-2'-hydroxyacetophenone semicarbazone (IX). This compound showed activity upto 73.0%
against C. falcatum_at 1000 ppm concentration.
Comparing the antifungal activity ofdifferent compounds can derive the following conclusions.
(a) There were significant alteration in the antifungal activity with the change in the nature of organic
group attached to O,O-diethylchlorophosphate moiety.
(b) For any particular species of fungus, organophosphorus derivatives containing substituted chalcones
were found to be more effective than its semicarbozone derivatives.
(c) For any particular series of organic compounds, the compounds containing chloro group in the
chalcone ring show better activity.
(d) The activity decreases ofdilution.
There results indicate that studies on organophosphorus derivations could be promising as fungicides
and constitute a new and promising field of application in disease and pest management.
Acknowledgement
One ofthe authors (OPP) is thankful to UGC, New Delhi for financial assistance.
References
1. C. Recaud, B.T. Eagan, A .G. Gillaspie. C.G. Hughes, Diseases of Sugarcane" Major Diseases,
Elsevier, Amsterdam, p. 399 (1989).
2. V.P. Agnihotri and A .K. Srivastava, Current Trends in sugarcane Pathology, International Books &
Periodicals Supply Service, Delhi, p. 235 (1994).
3. V.P. Agnihotri, Diseases of Sugarcane and Sugarbeet, Oxford & IBH, New Delhi, p 4 (1990).
4. G .P .Rao, P. Kumar, M. Singh, H.N.Singh, O .P. Pandey, Sugarcane, 17 (1994).
301
S.K. Sengupta et al. Efficacy ofOrganophosphorusderivatives ContaiTing Chalcones/
Chalcone Semicarbazones against Fungal Pathogens ofSugarcane
5. G .S. Gruzdyaev, V.A. Zinchenko, V.A. Kalinin, R.I. Slovtsov, The chennical protection of plants,
Mir, Moscoe, 1983.
6. K. Chaturvedi, O.P. Pandey and S .K. Sengupta, Synth. React. Inorg. Met.-org. Chem, 24 1487
(1994).
7. K. Chaturvedi S .K. Srivastava, O .P. Pandey and S .K. Sengupta, Synth React. hTorg. Met. -Org.
Chem., 25, 1191 (1995).
8. K. Chaturvedi, A .K. Jaiswai, O .P. Pandey and S .K. Sengupta, Synth. React. Inorg, Met-org Chem.,
25, 1581 (1995).
9. A.K. Jaiswal, G .P. Rao, O .P. Pandey and S .K. Sengupta, J. Agric. Food Chem., 46, 1609 (1998).
10. K. Chaturvedi, A .K .Jaiswal K.N. Mishra, O .P. Pandey and S .K. Sengupta, A.C.H. Models in
Chem., 135, 93 (1998).
11. S .K. Sengupta, O .P. Pandey, G.P.Rao, S .P. Shahi and A. K. Jaiswal, Sugarcane, 4, 17 (1998).
12. A .I. Vogel, A Text Books of practical organic Analysis ,3' Ed Longmans, London, 1956.
13. K. Chaturvedi, Ph. D, Thesis, University of Gorakhpur, 1993.
14. A .I. Vogel, A Text Books of Practical organic chemistry., 4tl'
Edn., Longmans London ), 1978.
15. R .K. Grover and .D. Moore, Phytopathology, 52, 876 (1962).
302

				

